# Factors associated with ocular injuries among adult road traffic accident patients presenting at Mulago National Referral Hospital, Uganda

**DOI:** 10.4314/ahs.v23i2.52

**Published:** 2023-06

**Authors:** Caroline Nalukenge, Francis O Sebabi, Bonny Okello, Jacob Ntende, Lydia Nakiyingi, Damalie Nakanjako, Faith Nakubulwa, Abubakar Kalinaki, Ben Mulinde, Anne A Musika

**Affiliations:** 1 Department of Ophthalmology, School of Medicine, College of Health Sciences, Makerere University, Kampala, Uganda; 2 Department of Economics and Statistics, Kyambogo University, Kampala, Uganda; 3 Department of Ophthalmology, Mulago National Referral Hospital, Kampala, Uganda; 4 Department of Medicine, School of Medicine, College of Health Sciences, Makerere University, Kampala, Uganda

**Keywords:** Ocular injury, road traffic accident, sub-Saharan Africa

## Abstract

**Background:**

Ocular trauma is the leading cause of unilateral blindness globally. Road traffic accidents are among the top risk factors for ocular trauma.

**Objectives:**

This study aimed to evaluate factors associated with ocular injuries among adult road traffic accident patients at Mulago Hospital, Uganda.

**Methods:**

A cross-sectional study was conducted among adult road traffic accident patients. History taking and ophthalmological examination were performed on consenting participants. Data was analysed using STATA version 14.0.

**Results:**

Overall, 428 road traffic accident cases were enrolled, of which majority (84.3%) were male. Age 30-39years (aOR = 0.58, 0.36 – 0.94, p = 0.027), being male (aOR = 2.64, 1.21 – 5.13, p = 0.004) and being a passenger of motor vehicle/cycle (aOR = 3.85, 1.49 – 9.93, p = 0.005) were the factors associated with ocular injuries among the participants.

**Conclusions:**

Age 30-39 years, male gender and being a passenger of motor vehicle/cycle were the factors associated with ocular injuries among the adult road traffic accident patients. Ocular injuries were more common among the road users who did not use safety measures. Use of safety measure by passengers of motor vehicles and cycles is recommended.

## Introduction

More than 1 million people worldwide have bilateral visual loss due to trauma while 19 million people have monocular blindness, making ocular trauma the leading cause of unilateral blindness 1. Road traffic accidents (RTA) are among the top risk factors for ocular trauma 2. There is a global increase in the number of RTAs particularly in the developing countries 3. The factors that contribute to the increased occurrence of RTAs in many developing countries include bad roads, poor vehicle conditions, reckless driving, driving under the influence of alcohol or other drugs and poor adherence to traffic rules 4 5.

In Uganda's capital city, Kampala, a study done found that RTAs were the most common cause of injuries, contributing 50% of all the injuries in Kampala 5. At Mulago hospital, Uganda's national referral Hospital, RTAs are the leading cause of surgical admission and patients present mostly with lower extremity injuries followed by head injuries, who are most likely to have ocular injuries 6.

There is limited data on factors associated with ocular injuries among RTA patients in Uganda. Understanding the factors associated with ocular injuries among RTAs will guide in policy making towards prevention of ocular injuries among RTA patients in Uganda and the region. We therefore evaluated the factors associated with ocular injuries among adult road traffic accident patients at MNRH, Kampala, Ganda.

## Methods

### Study design and setting

A hospital-based, cross-sectional study was conducted at the Mulago National Referral Hospital (MNRH) emergency department, from January 2020 to March 2020. MNRH is the national referral hospital in Uganda, located on Mulago Hill in the northern part of the city of Kampala, immediately west of the Makerere University College of Health Sciences (Mak-CHS), a teaching facility for Mak-CHS and the largest public hospital in Uganda. It is approximately 5 kilometres by road, north-east of Kampala's central business area. The hospital's emergency department has a bed capacity of 25 and receives a relatively high number of RTA patients, on average about 15 RTA survivors per day.

### Study participants

Every second adult RTA patient, 18 years and above, was enrolled into the study until the required sample size was achieved. All study participants provided written informed consent.

### Study procedure

After obtaining written informed consent, the study doctor took the patient's history and performed an ophthalmological examination on all patients which included visual acuity using a Snellen's chart, torch light examination, slit lamp examination using a portable slit-lamp, intraocular pressure (IOP) measurement, and fundoscopy where possible, to look for presence of ocular injuries. Ocular ultrasound scan and Computerised Tomography (CT) scan, were done whenever indicated by the examining clinician.

### Data management and statistical analysis

Data were collected using a standardised semi-structured questionnaire which was checked for completeness, entered in Epi data version 3.1 and analysed using STATA version 14.0. Continuous variables were summarised using means and standard deviations. Categorical variables were summarised using frequencies, proportions and percentages. Data was presented using tables.

Logistic regression was performed at bivariate level for each of the independent variables to determine how they were independently associated with ocular trauma. Variables with a p-value of < 0.2 at bivariate analysis were considered for multivariate analysis. We assessed for confounders putting into consideration multi-collinearity between the variables. Significance was set at p-value of 0.05 or less.

### Ethical considerations

Approval for this study was granted by the School of Medicine Research and Ethics Committee for the College of Health Sciences. All participants gave informed written consent.

## Results

### Demographic factors and ocular injuries of the RTA patients

In the bivariate logistic regression, age, gender, marital status, education level and occupation were analysed for association with ocular injuries among RTA patients. Patients aged 30-39 years and being a male RTA patient were statistically significantly associated with ocular injuries at p < 0.05 and 95% confidence interval. However, marital status, education level and occupation were not statistically significantly associated with ocular injuries as shown in table 1.

**Table 1:** Bivariate logistic regression of demographic factors and ocular injuries among adult patients at MNRH emergency department

Variable (n=428)	Frequency (%)	Ocular Trauma present (n=148) (f, %)	Ocular Trauma Absent (n=280) (f, %)	p value
**Age category**				
>50	21(4.9)	7(4.7)	14(5.0)	0.491
40-49	46(10.8)	15(10.1)	31(11.1)	0.289
30-39	164(38.3)	45(30.5)	119(42.5)	**0.007** *
18-29	197(46)	81(54.7)	116(41.4)	
**Sex**				
Male	361(84.3)	134(90.5)	227(81.1)	**0.011** *
Female	67(15.7)	14(9.5)	53(18.9)	
**Marital status**				
Married	226 (52.8)	80(54.1)	146(52.1)	0.884
Separated	9(2.1)	2(1.4)	7(2.5)	0.446
Single	193 (45.1)	66(44.6)	127(45.4)	
**Education level**				
Tertiary	77(17.9)	18(12.2)	59(21.1)	0.333
Secondary	181(42.2)	67(45.3)	114(40.7)	0.923
Primary	156(36.6)	58(39.2)	98(35.0)	0.913
None	14(3.3)	5(3.4)	9(3.2)	
**Occupation**				
Employed	321(75)	108(73.0)	213(76.1)	0.482
Not employed	107(25)	40(27.0)	67(23.9)	

* *Denotes statistical significance at 95% confident interval and p-value <0.05*.

### Behavioral factors and ocular injuries among adult RTA patients

The RTA patients who presented to MNRH emergency unit had varied behavioral factors before and at the time of the accident. These factors were assessed for association using bivariate analysis. It was found that, being a passenger and failure to use road safety measures were statistically significantly associated with ocular injuries among adult RTA patients at MNRH emergency unit 95% confident interval and p<0.05. The factors such as alcohol use, under influence of alcohol at the time of accident, being pedestrian, motor cyclist or driver and use of safety measures were not statistically significantly associated with ocular injuries. These findings are shown in table 2.

**Table 2:** Bivariate logistic regression of behavioral factors and ocular injuries among RTA patients at MNRH emergency department

Variable (n=428)	Frequency (%)	Ocular Trauma present (n=148) (f, %)	Ocular Trauma Absent (n=280) (f, %)	p value
**Alcohol use**				
Yes	142(33.2)	46(31.1)	96(34.3)	0.459
No	286(66.8)	102(68.9)	184(65.7)	
**Under the influence alcohol prior to the accident (n=142)**				
Yes	53(37.3)	20(43.5)	33(34.4)	0.295
No	89(62.7)	26(56.5)	63(65.6)	
**Type of road-user**				
Pedestrian	102(23.8)	33(22.3)	69(24.6)	0.369
Passenger	42(9.8)	23(15.5)	19(6.8)	**0.004** *
Motor cyclist	229(53.5)	78((52.7)	151(53.9)	0.223
Driver	55(12.9)	14(9.5)	41(14.6)	
‡**Safety measure use (n=326)** ^a^				
Yes	145(44.5)	25(21.7)	120(56.9)	
No	181(55.5)	90(78.3)	91(43.1)	**0.000** *
**Type of safety measure (n=145)**				
Helmet	96(66.2)	16(64.0)	80(66.7)	0.798
Seatbelt	49(33.8)	9(36.0)	40(33.3)	

* *Denotes statistical significance at 95% confident interval and p-value <0.05*.

a *102 participants were pedestrians for whom the safety measures in question were not applicable*

‡ *Safety measure used was not considered in multivariate logistic regression because it was collinear with type of road-user and yet excluded pedestrians hence would affect the outcome for type of road user at multivariate analysis*.

RTA patients aged 30-39 years, male gender and being a passenger were subsequently analysed using multivariate logistic regression to offset the confounders. The factors that were found to be associated with ocular injuries among adult RTA patients using multivariate logistics regression were patients aged 30-39 years (aOR = 0.58, 0.36 – 0.94, p = 0.027), male gender (aOR = 2.64, 1.21 – 5.13, p = 0.004) and being a passenger (aOR = 3.85, 1.49–9.93, p = 0.005) as indicated in table 3.

**Table 3:** Multivariate logistic regression for factors associated with ocular injuries among adult RTA patients at Mulago hospital emergency department

Variable (n=428)	cOR and 95% Confidence Interval	p value	aOR and 95% Confidence Interval	p value
**Age category**				
≥50	0.72(0.28,1.85)	0.491	0.71(0.26,1.95)	0.509
40-49	0.69(0.35,1.37)	0.289	0.70(0.34,1.46)	0.347
30-39	0.54(0.35,0.85)	0.007	0.58(0.36,0.94)	**0.027** *
18-29	1.00		1.00	
**Gender**				
Male	2.26(1.21,4.23)	0.011	2.64(1.36,5.13)	0.004*
Female	1.00		1.00	
**Occupation**				
Employed	0.85(0.54, 1.34)	0.482	0.95(0.57,1.58)	0.847
Unemployed	1.00		1.00	
**Type of road-user**				
Pedestrian	1.40(0.67,2.92)	0.369	1.45(0.64,3.27)	0.372
Passenger	3.55(1.50,8.37)	0.004	3.85(1.49,9.93)	**0.005** *
Motor cyclist	1.51(0.78,2.94)	0.223	1.37(0.66,2.83)	0.394
Driver	1.00		1.00	
**Under influence of alcohol**				
Yes	1.17(0.65,2.12)	0.606	1.52(0.80,2.87)	0.200
No	1.00		1.00	

* *Denotes statistical significance at p-value <0.05, cOR=Crude Odds Ratio, aOR=Adjusted Odds Ratio*

### Participants' characteristics and types of road users among adult RTA patients who had ocular injuries

RTA patients aged 18-29 were the majority among the road users with an exception of the drivers. Likewise, among different road users, male road users were more dominant compared to the female counterparts. Drivers used safety measures more than any other types of road users. Twenty-two (95.6%) of the passengers did not use safety measures as shown in the table 4.

**Table 4:** Pearson's Chi- squared correlation between participants' characteristics and types of road users among adult RTA patients who had ocular injuries at MNRH emergency department

Variable (n=428)	Pedestrian (n=33) (n, %)	Motorcyclist (n=78) (n, %)	Drivers (n=14) (n, %)	Passenger (n=23) (n, %)	P value
**Age**					
18-29 years	22(66.7)	45(57.7)	0(0.00)	15(65.2)	0.000*
30-39 years	9(27.2)	26(33.3)	8(57.1)	4(17.4)	
40-49 years	2(6.1)	6(7.8)	2(14.3)	4(17.4)	
≥50 years	0(00)	1(1.2)	4(28.6)	0(00)	

**Sex**					
Male	26(78.8)	75(96.2)	13(92.9)	20(86.9)	0.035*
Female	7(21.2)	3(3.8)	1(7.1)	3(13.1)	

**Use of safety measures**					
Yes	-	16(20.5)	8(57.1)	1(4.4)	0.001*
No	-	62(79.5)	6(42.9)	22(95.6)	

### Types of ocular injuries and use of road safety measures

The commonest ocular injuries were eyelid periorbital edema and ecchymosis, sub-conjunctival hemorrhage, eyelid partial thickness laceration and conjunctival chemosis. These ocular injuries were the commonest in both groups of RTA patients who did not use and those who used road safety measures. However, ocular injuries were generally more common among the road users who did not use safety measures compared to those who used the measures as indicated in table 5.

**Table 5:** Types of ocular injuries and use of road safety measures among adult RTA patients at MNRH emergency department

Type of Ocular injuries (n=115)	Use of road safety measures	
	No (n=90) f (%)	Yes (n=25) f (%)
Eyelid periorbital edema and ecchymosis	68 (75.5)	19 (76.0)
Sub-conjunctival hemorrhage	58 (64.4)	15 (60.0)
Eyelid partial thickness laceration	31 (34.4)	8 (32.0)
Chemosis	22 (24.4)	3 (12.0)
Orbital fracture	12 (13.3)	2 (8.0)
Corneal oedema	11 (12.2)	2 (8.0)
Eyelid bruises	5 (5.5)	4 (16.0)
Eyelid full thickness laceration	6 (6.7)	1 (4.0)
Hyphaema	6 (6.7)	0 (0.0)
Vitreous hemorrhage	6 (6.7)	0 (0.0)
Traumatic mydriasis	5 (5.5)	0 (0.0)
Whole globe rupture	3 (3.3)	0 (0.0)
Lens Cataract	3 (3.3)	0 (0.0)
Scleral partial thickness wound	2 (2.2)	0 (0.0)
Scleral perforation	1 (1.1)	0 (0.0)
Extra ocular muscle entrapment	1 (1.1)	0 (0.0)
Corneal epithelial defect	1 (1.1)	0 (0.0)
Trauma optic neuropathy	1 (1.1)	0 (0.0)

*Denotes statistical significance at 95% confident interval and p-value <0.05.

## Discussions

Our study found that majority (46%) of the RTA patients were aged 18-29years and 84.3% were male with a male to female ratio of 5:1. These findings are similar to the findings of a study done in India among RTA patients which reported that majority (35.86%) of the RTA patients were aged 21-30years and 82.86% were male with a male to female ratio of 5:1 7. Another study done in India equally found that the maximum number (34.02%) of RTA cases were aged 21-30years 8. Akinpelu et al explained that young adult males are more prone to road traffic injuries because they are engaged in a wide range of outdoor activities hence have more reason to move from place to place 4.

We found that age 30-39years (aOR = 0.58, 0.36 - 0.94, p = 0.027), being male (aOR = 2.64, 1.21 - 5.13, p = 0.004) and being a passenger of motor vehicle/cycle (aOR = 3.85, 1.49 - 9.93, p = 0.005) were the factors associated with ocular injuries among the adult RTAs at Mulago National Referral Hospital emergency unit.

In our study, adult RTA patients aged 30-39 years were 42% less likely to have ocular injuries than those aged 18-29 years. Das et al in their study, found that majority of the ocular trauma cases following RTAs were in the age group of 21-30 years 9. This could be because young adults are the productive age group and are therefore more involved in the outdoor activities. They are also more involved in risky road behavior, which could make them more prone to RTAs and their associated ocular injuries.

Present study also found that male gender was 2.6 times more likely to present with ocular injuries following RTA than their female counterparts. Studies in India reported a similar finding of male preponderance among RTA patients with ocular injuries 9, 10. A study in Nigeria attributed the male preponderance of ocular injuries to the fact that males exhibit greater mobility and high-risk behaviour hence prone to reckless driving and increased liability to getting involved in RTAs 11. Namala et al, in their study, explained that females are less prone to RTA-related ocular injuries because they are confined to home and do less risky jobs than males 12.

Our study found that passengers were almost 4 times more likely to present with ocular injuries than the drivers. This could be because almost all the passengers (95.6%) reported not to have used safety measures prior to the accident. In assessing the ocular injuries among road users who had used safety measures and those who had not, our study found that road users who had not used safety measures prior to the accident were more likely to get ocular injuries compared to their counterparts. Present study also found that more severe injuries such as hyphaema, vitreous hemorrhage, globe ruptures, lens cataract, traumatic optic neuropathy were only seen among road users who had not used safety measures prior to the accident. This implies that using safety measures could protect RTA patients from sustaining ocular injury or severe sight-threatening ocular injuries. A study done in USA reported a two-fold reduction in eye injuries following seatbelt use 13. Dawson et al recommended that better implementation of traffic rules, wearing helmets with two-wheeler driving, and observance of other traffic rules could minimize morbidity due to ocular trauma 14.

## Study limitations

Our study being a hospital based cross section study, has limitation in generalizability to RTA patients who did not come to MNRH emergency unit for clinical evaluation. Some RTA patients who presented to MNRH emergency unit could have had non-RTA ocular injuries prior to the accident. Our study being a cross-sectional study, ocular injuries found during this season may not be the same in other seasons since RTAs tend to increase in the rainy season. RTA patients who presented at night or weekends and discharged, could have been missed out.

## Conclusions and recommendations

Age 30-39 years, male gender and being a passenger of motor vehicle/cycle were the factors associated with ocular injuries among the road traffic accident (RTA) at Mulago National Referral Hospital emergency unit. Ocular injuries were generally more common among the road users who did not use safety measures compared to those who used the measures. Sight-threatening ocular injuries such as hyphaema, cataract, vitreous hemorrhage and traumatic optic neuropathy were only found among the road users who had not used safety measures. We therefore recommend use of road safety measures such as seatbelts and helmets to be enhanced among passengers of motor vehicles and cycles.
